# Optic Nerve Sheath Diameter for Predicting Outcomes in Post-Cardiac Arrest Syndrome: An Updated Systematic Review and Meta-Analysis

**DOI:** 10.3390/jpm12030500

**Published:** 2022-03-20

**Authors:** Jae-Guk Kim, Wonhee Kim, Hyungoo Shin, Tae-Ho Lim, Bo-Hyoung Jang, Youngsuk Cho, Kyu-Sun Choi, Min-Kyun Na, Chiwon Ahn, Juncheol Lee

**Affiliations:** 1 Department of Emergency Medicine, College of Medicine, Hallym University, Chuncheon 24253, Korea; gallion00@gmail.com (J.-G.K.); wonsee02@gmail.com (W.K.); faith2love@hanmail.net (Y.C.); 2 Department of Emergency Medicine, Hanyang University College of Medicine, Seoul 04763, Korea; erthim@gmail.com (T.-H.L.); jclee0221@gmail.com (J.L.); 3 Department of Preventive Medicine, College of Korean Medicine, Kyung Hee University, Seoul 02447, Korea; bhjang@khu.ac.kr; 4 Department of Neurosurgery, Hanyang University College of Medicine, Seoul 04763, Korea; vertex-09@hanmail.net (K.-S.C.); mavmav@hanmail.net (M.-K.N.); 5 Department of Emergency Medicine, College of Medicine, Chung-Ang University, Seoul 06974, Korea; cahn@cau.ac.kr

**Keywords:** optic nerve sheath diameter, heart arrest, patient outcome assessment, meta-analysis

## Abstract

We aimed to identify the efficacy of optic nerve sheath diameter (ONSD) in predicting mortality and poor neurological outcomes (PNO) in post-cardiac arrest syndrome (PCAS) by the measurement time of outcomes. We conducted an extensive literature search in EMBASE, MEDLINE, and Cochrane Library, which included studies on the prognostic accuracy of ONSD in predicting PNO and mortality in PCAS by the measured time of outcomes. A total of 791 patients from nine studies were included. Increased ONSD was weakly associated with PNO by a high heterogeneity (standardized mean difference with 95% confidence interval = 0.74 (0.22, 1.27); I^2^ = 87%). The analysis by the measurement time of PNO and mortality for ONSD had no significant difference due to insufficient articles or high heterogeneities. The prognostic accuracy of ONSD was 23.97 (pooled diagnostic odds ratio, I^2^ = 0%) and 0.94 (area under the curve) for short-term PNO. The pooled results showed low or very low quality and very low quality of evidence for PNO and mortality, respectively. ONSD measurement might be an effective predictor for short-term PNO in PCAS. An analysis by measurement time of outcomes showed no significant evidence for ONSD measurement effectiveness in predicting mortality and PNO.

## 1. Introduction

According to cited literature, optic nerve sheath diameter (ONSD) measurement has been used for the early prediction of patient outcomes in post-cardiac arrest syndrome (PCAS) [1,2]. ONSD can be measured after achieving a return of spontaneous circulation (ROSC) using several imaging tools, such as brain computed tomography (CT), magnetic resonance imaging (MRI), and bedside ocular ultrasonography (US) [1,3,4].

ONSD measurement is useful as a prognostic predictor in patients with PCAS. This is because dilated ONSD is strongly associated with increased intracranial pressure (ICP) and brain edema [3,5,6]. When hypoxic and ischemic insults from cardiac arrest (CA) cause brain edema and increase ICP, ONSD increases by shunting cerebrospinal fluid (CSF) into the optic nerve sheath [1,3,6].

Using ocular US and brain CT, two recent systematic reviews demonstrated that ONSD had a high diagnostic value for predicting poor neurological outcomes (PNO) in patients with PCAS [7,8]. In these reviews, ONSD showed low pooled sensitivity, high pooled specificity, and high area under the receiver-operating characteristic curve. However, these reviews have some significant weaknesses for inclusion in meta-analyses. These weaknesses include heterogeneous definitions for PNO (cerebral performance category (CPC) 5 or 3–5) and a wide range of outcome measurement time points (at hospital discharge, 1 month, 3 months, and 6 months). We hypothesized that these heterogenic issues regarding outcomes can result in an inaccurate diagnostic performance of ONSD. Moreover, the prognostic value of ONSD in PCAS remains controversial.

We conducted a systematic review and meta-analysis to identify the prognostic efficacy of ONSD for mortality and PNO in PCAS by measurement time.

## 2. Materials and Methods

### 2.1. Protocol and Registration

This systematic review and meta-analysis was conducted according to the Preferred Reporting Items for Systematic Reviews and Meta-Analyses of Diagnostic Accuracy Studies statement [9]. The review protocol was registered with PROSPERO (http://www.crd.york.ac.uk/PROSPERO/, accessed on 29 January 2021), with registration number CRD42021234088.

### 2.2. Eligibility Criteria

The population, intervention, comparison, and outcome (PICO) clinical question was applied in this study. We performed a literature search and selected eligible studies. The study outcomes were then evaluated in a meta-analysis. The PICO question was as follows: population (P) = adult PCAS; intervention (I) = ONSD; comparator (C) = none; outcome (O) = PNO and in-hospital mortality.

### 2.3. Information Sources and Literature Search Strategy

We performed an extensive database search for all relevant studies that examined the effectiveness of ONSD in predicting neurological outcomes and in-hospital mortality in patients with PCAS. The literature search was performed in the EMBASE (from 1974 to 14 June 2021) and MEDLINE (from 1946 to 14 June 2021) databases through the Ovid interface and the Cochrane Library (all years). The latest date for updating our search was set by two experienced reviewers (J. Kim and W. Kim) on 17 June 2021. The following search terms were used: “cardiac arrest”, “heart arrest”, “cardiopulmonary resuscitation”, “return of spontaneous resuscitation”, “advanced cardiac life support”, and “optic nerve sheath diameter” (Table S1). Additionally, we manually checked the references of eligible studies to identify other relevant studies. No language restrictions or methodology filters were used, and all prospective or retrospective observational studies were included.

### 2.4. Study Selection

Two experienced reviewers (J.-G. Kim and W. Kim) independently screened the titles and abstracts of all eligible studies to exclude irrelevant studies. Studies were excluded if they met the following criteria: duplicate studies, irrelevant populations, irrelevant comparisons, irrelevant outcomes, reviews, case reports, editorials, letters, comments, conference abstracts, animal studies, and meta-analyses. In case of disagreement between the two reviewers, a third reviewer (H. Shin) was consulted, and differences were resolved through discussion until a consensus was reached. After excluding ineligible abstracts, the full texts of the selected studies were rescreened and reviewed thoroughly for eligibility using the predetermined selection criteria. Studies with insufficient data, despite contacting the authors for further details, were also excluded.

### 2.5. Data Collection Process and Data Items

Two reviewers (J.-G. Kim and W. Kim) independently extracted information on the basic characteristics and main results of the selected studies. Any disagreement between reviewers was resolved through discussion with a third reviewer (H. Shin). Study characteristics and the extracted covariates were summarized using standard descriptive statistics. Dichotomous variables were reported as frequencies (%), whereas continuous variables were reported as means (standard deviation (SD)). Data on the following variables were extracted: author, year of publication, country, inclusion period, study type, sample size, type of CA (out-of-hospital CA, OHCA vs. in-hospital CA, IHCA), number of patients with PNO and in-hospital mortality, modality and method used for ONSD measurement, time from ROSC to ONSD measurement, tool for neurological outcomes (CPC or Glasgow outcome scale (GOS)), and time points of outcome measurement. The neurological outcome was classified as either good or poor, based on the CPC (1–2 = GNO; 3–5 = PNO) and GOS (4–5: GNO; 1–3: PNO) scores. The mean (±SD) ONSD of patients or the estimated mean (±SD) values were calculated from the median values with interquartile ranges using the method devised by Wan et al. [10].

To identify the prognostic accuracy of ONSD in predicting PNO in PCAS, we generated 2 × 2 tables with two variables for ONSD and the neurological outcome. Data regarding true-positive (TP), false-positive (FP), false-negative (FN), and true-negative (TN) results for individual studies were also obtained. The 2 × 2 tables in the included studies were presented as follows: TP = PNO with increased ONSD, FP = GNO with increased ONSD, FN = PNO with no increase in ONSD, and TN = GNO with no increase in ONSD. If none of these variables were described in the studies, we requested further details from the corresponding author of each study through email.

### 2.6. Risk of Bias in Individual Studies

Two reviewers (J.-G. Kim and W. Kim) independently estimated the methodological quality of the included studies, blinded to authorship and journal details. Patient selection, index test, reference standard, flow, and timing were assessed using a revised quality assessment of diagnostic accuracy studies (QUADAS-2) tool [11].

### 2.7. Statistical Analysis

In the main analysis, we estimated the association between ONSD and PCAS outcomes. The strength of the association between increased ONSD and poor outcomes (PNO and in-hospital mortality) was estimated using standardized mean differences (SMD). The ONSD across comparison groups was determined and presented as mean differences with 95% confidence intervals (CIs). A random-effects model was used to synthesize the individual data of the included studies considering the diversity of countries, inclusion periods, modalities for ONSD measurement, and time points of outcome measurement. To measure heterogeneity, I^2^ statistics were used to estimate the between-study inconsistency proportion due to true differences between studies (rather than differences due to random error or chance), with values of 25%, 50%, and 75% being considered low, moderate, and high, respectively [12].

Additionally, we attempted to identify the prognostic accuracy of ONSD in predicting PNO in patients with PCAS. Pooled sensitivities, specificities, positive and negative likelihood ratios, and diagnostic odds ratios (DORs) were calculated by generating 2 × 2 tables with two variables for ONSD and the neurological outcome. Summary receiver-operating characteristic curves were constructed and used to summarize the overall prognostic performance of ONSD, which also represented the calculated value of the Q* index and area under the characteristic curve (AUC) [13,14]. AUC values were assessed using the following categories: more than 0.97 (excellent), from 0.93 to 0.96 (very good), from 0.75 to 0.92 (good), and less than 0.75 (reasonable but obviously deficient in prognostic accuracy) [15].

We performed planned subgroup analyses for the following confounders: location details (country (Korea vs. other countries)); PNO (>65% vs. <65%) according to the median value across the included studies; modality used for ONSD measurement (CT vs. US); proportion of targeted temperature management (TTM) (100% vs. <100%). To identify the potential causes of heterogeneity, sensitivity and meta-regression analyses were performed using the following covariates: sample size, time from ROSC to ONSD measurement, mean age, proportion of males and shockable rhythm. 

The reference management software Endnote X9 (Clarivate Analytics, Philadelphia, PA, USA) was used to organize all identified studies in the literature search. We used Meta-Disc software, version 1.4 (Clinical Biostatistics, Ramony Cajal Hospital, Madrid, Spain), and RevMan, version 5.3 (Cochrane Collaboration, Oxford, UK), to perform statistical analyses, and *p* < 0.05 was considered statistically significant.

The meta-regression analysis was performed and the risk of bias across studies was assessed using the R package “meta” (R, version 3.3.2; R Foundation for Statistical Computing, Vienna, Austria). Publication bias was assessed using funnel plots and the Egger’s test. The asymmetry of the funnel plot and *p* < 0.05 using Egger’s test indicated the presence of bias. We used the GRADEpro Guideline Development Tool (McMaster University and Evidence Prime, Inc., Hamilton, Ontario, Canada) to evaluate the quality of evidence of each study. Evidence was summarized according to GRADE levels (high, moderate, low, and very low) through evaluating design, risk of bias, consistency, precision, directness, and possible publication bias of the included studies.

## 3. Results

### 3.1. Study Selection

**Figure 1:**
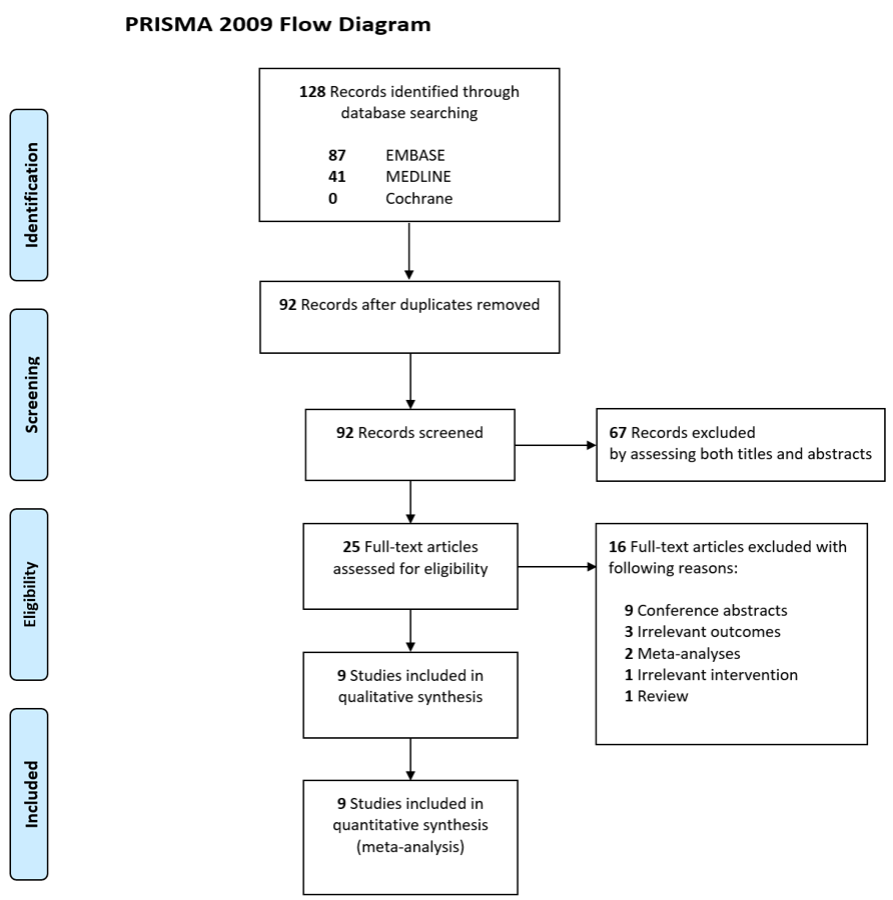
Flow diagram of studies included in the meta-analysis.

A flow diagram of the literature search for this systemic review is shown in Figure 1. Overall, 128 records were identified using the database search. Thirty-six duplicate records were removed, and 67 additional irrelevant records were excluded after screening the titles and abstracts. After the full texts of the 25 remaining records were reviewed, we excluded 16 records for reasons such as conference abstracts (*n* = 9), irrelevant outcome measures (*n* = 3), meta-analyses (*n* = 2), irrelevant intervention (*n* = 1), and reviews (*n* = 1). Finally, nine observational studies that enrolled a total of 791 patients were included in the meta-analysis [1,3,6,16,17,18,19,20,21].

### 3.2. Study Characteristics

The main attributes of the included studies are listed in Table 1. Additionally, baseline characteristics of the enrolled patients are provided in Table S2. Nine observational studies were published during 2014–2019 [1,3,6,16,17,18,19,20,21]. Five studies were conducted in Korea [1,17,18,19,20], whereas the other studies were conducted in Canada, Europe, and Japan [3,6,16,21]. Six studies were single-center studies [1,6,17,19,20,21], and three were multicenter studies [3,16,18]. ONSD was measured using CT in five studies [1,6,17,18,20] and US in four studies [3,16,19,21]. All studies measured the ONSD at 3 mm behind the globe, and ONSD was measured within 72 h from ROSC [1,3,6,16,17,18,19,20,21].

Seven studies had neurological outcomes as the main outcome measurements [1,6,17,18,19,20,21]. On the measurement time of outcomes, there were three studies at hospital discharge, two studies at 1 month, one study at 3 months, and one study at 6 months. Five studies reported mortality as a patient outcome [3,6,16,18,20]: two studies at hospital discharge and one study at 6 months.

Six studies assessed neurological outcomes according to the CPC and considered a CPC score of 3–5 as a PNO [1,6,17,18,19,20]. One study evaluated patients using the GOS, and a score of 1–3 was considered as a PNO [21]. The proportion of patients with a PNO was 63.2% in seven studies [1,6,17,18,19,20,21], while mortality was 50.5% in five studies [3,6,16,18,20].

### 3.3. Risk of Bias within Studies

The risk of bias and applicability concerns for the nine included studies were assessed using the QUADAS-2 tool (Figure S1). The risk of bias for the index test was found to be high in four studies. All included studies exhibited low applicability concerns in terms of patient selection, index test, and reference standard domains.

### 3.4. Quality of Evidence According to GRADE Levels

The included studies were found to have a low quality or very low quality of evidence for PNO and very low quality of evidence for death (Table S8).

### 3.5. Results of Meta-Analyses

#### 3.5.1. ONSD as a Predictor for PNO

**Figure 2:**
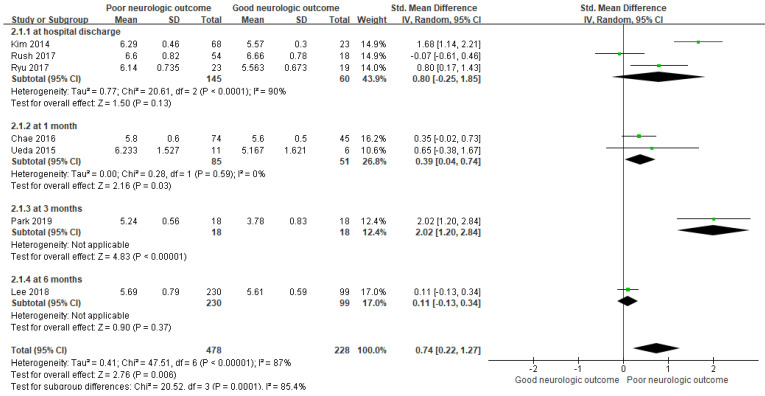
Forest plot for the association of ONSD with a poor neurological outcome. Abbreviations: CI = confidence interval; ONSD = optic nerve sheath diameter; SD = standard deviation.

In this meta-analysis, ONSD was found to be significantly increased in the PNO group compared to that in the GNO group, demonstrating a positive association with an overall SMD ((mean value in the PNO group—mean value in the GNO group)/pooled SD) of 0.74 (95% CI = 0.22–1.27; I^2^ = 87%; *p* = 0.006; Figure 2). There was no significant difference in ONSD as a predictor of neurological outcomes at the time of hospital discharge between the PNO and GNO groups (3 studies; SMD = 0.80; 95% CI = −0.25–1.85; I^2^ = 90%; *p* = 0.13).

#### 3.5.2. ONSD for Predicting Mortality

**Figure 3:**
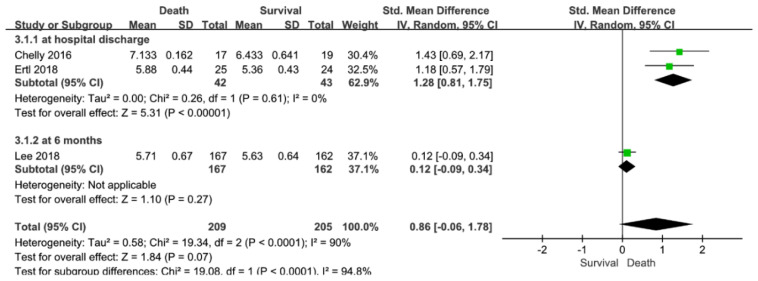
Forest plot for the association of ONSD with death. Abbreviations: CI = confidence interval; ONSD = optic nerve sheath diameter; SD = standard deviation.

There was no significant difference in ONSD between survival and death (3 studies; SMD = 0.86; 95% CI = −0.06–1.78; I^2^ = 90%; *p* = 0.07; Figure 3).

#### 3.5.3. Prognostic Accuracy of ONSD in Predicting PNO

Five studies were included in summary estimates with the prognostic accuracy of ONSD in predicting PNO [1,17,19,20,21]. All studies were single-center studies conducted in East Asia (4 in Korea and 1 in Japan). ONSD was measured using CT in three studies [1,17,20] and US in two studies [19,21]. The total number of patients was 305, and the proportion of patients with PNO was 63.6%. The time point of neurological outcomes measurement ranged from hospital discharge to 3 months.

**Figure 4:**
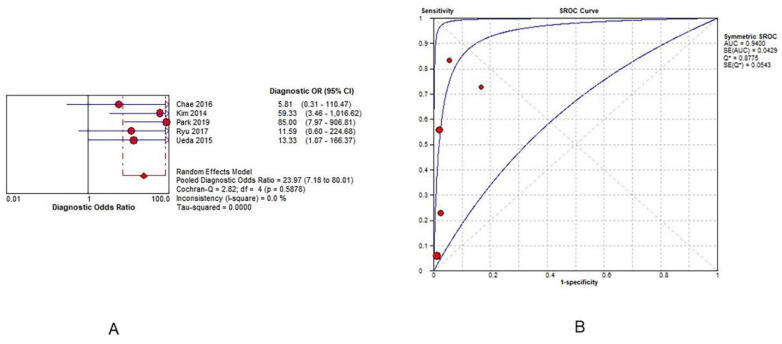
Prognostic accuracy of ONSD for a poor neurological outcome. (**A**): diagnostic odds ratio of ONSD in predicting a neurological outcome; (**B**): SROC curve of ONSD in predicting a neurological outcome. Abbreviations: CI = confidence interval; ONSD = optic nerve sheath diameter; SD = standard deviation; SROC = summary receiver-operating characteristic.

The pooled DOR value of ONSD for predicting PNO was 23.97 (95% CI = 7.18–80.01; I^2^ = 0%). The AUC value was 0.94 (standard error = 0.04; Q = 0.87) in five studies (Figure 4). The sensitivity and specificity of ONSD ranged from 0.05 to 0.83 and 0.83 to 1.00, respectively (Table S3). The pooled sensitivity and specificity were 0.36 (95% CI = 0.29–0.43; I^2^ = 94.7%) and 0.98 (95% CI = 0.94–1.00; I^2^ = 42.0%), respectively. The pooled positive and negative likelihood ratios were 9.10 (95% CI = 3.26–25.40; I^2^ = 0) and 0.50 (95% CI = 0.25–1.00; I^2^ = 96.9), respectively (Figure S2).

#### 3.5.4. Risk of Bias across Studies

**Figure 5:**
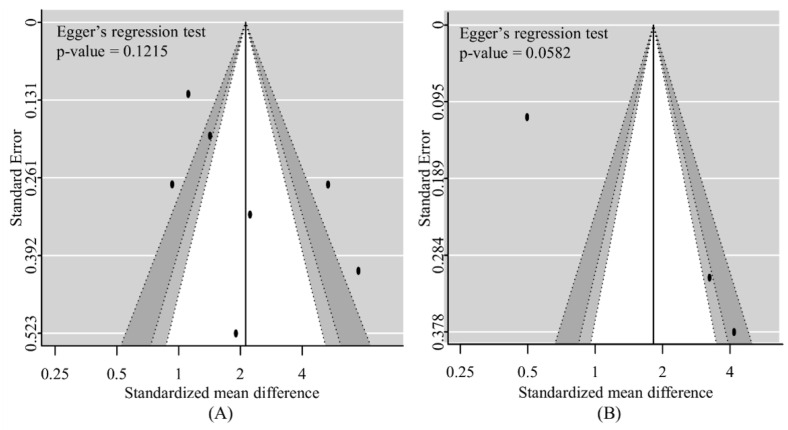
Funnel plot assessing publication bias for a poor neurological outcome and death. (**A**): Publication bias for a poor neurological outcome; (**B**): Publication bias for death.

There was no definite asymmetry in the forest plots. No significant publication bias existed statistically in the assessment based on the Egger’s regression test with studies for predicting neurological outcomes (*p* = 0.1215) and death (*p* = 0.0582) (Figure 5).

#### 3.5.5. Additional Analyses

The results of the subgroup and sensitivity analyses are provided in Tables S4 and S5, respectively. There were no significant differences in heterogeneity. The results of the meta-regression analyses are presented in Table S6. No significant association was identified between the neurological outcome and sample size, time from ROSC to ONSD measurement, mean age, proportion of males, and proportion of shockable rhythm.

## 4. Discussion

In this meta-analysis, the summary estimates of the sensitivity and specificity of ONSD in predicting PNO were 0.36 (95% CI = 0.29–0.43; I^2^ = 94.7%) and 0.98 (95% CI = 0.94–1.00; I^2^ = 42.0), respectively. The pooled DOR value of ONSD in PCAS was 23.97 (95% CI = 7.18–80.01; I^2^ = 0%), and the AUC value was 0.94. In the analysis of ONSD as a predictor of neurological outcomes, the overall ONSD was found to be higher in the PNO group than in the GNO group (SMD = 0.74; 95% CI = 0.22–1.27; I^2^ = 87%). However, unlike previous systematic reviews that showed usefulness in predicting neurological prognosis of ONSD [7,8], it is difficult to determine whether an enlarged ONSD is useful for the prediction of neurological outcomes in PCAS. This is because after separating the neurological outcome by the measurement interval, we found that the results were different depending on the measurement time and that the low heterogeneity of outcomes (1 month and in-hospital mortality) was not feasible to draw a conclusion, owing to the lack of studies and low quality of evidence according to GRADE level.

The optic nerve is surrounded by an optic nerve sheath, which is filled with CSF and communicates directly with the subarachnoid space. The ONSD reflects elevated ICP due to the inflation of the optic nerve sheath with the CSF [7,8,22,23,24]. In PCAS, an elevated ICP is correlated with neurological prognosis [7,25,26]. Therefore, the ONSD measurement could be crucial in predicting the outcome following PCAS [6,27,28].

In two previous meta-analyses conducted by Lee et al. and Zhang et al. [7,8], ONSD was reported to be useful in predicting neurological outcomes in PCAS. These meta-analyses had several limitations, such as heterogeneous criteria for PNO (CPC 5 or CPC 3–5) and a wide range of outcome measurement time points, ranging from hospital discharge to 6 months. Furthermore, Lee et al. reported that ONSD had a sensitivity of 41% and a specificity of 99% for the prediction of neurological outcomes in patients with PCAS. However, these two values also had considerable heterogeneity in both sensitivity (I^2^ = 72.3%) and specificity (I^2^ = 94.60%) [7]. In the correspondence by Huang et al. [29], they noted incorrect pooling in the meta-analysis by Lee et al. [7] An included study did not report the cut-off, sensitivity, and specificity of ONSD, which means that data extraction for correct pooling was statistically impossible [18]. Additionally, Huang et al. [29] noticed that another included study, which was conducted by Lee et al. [7] but not included in our meta-analysis for neurological outcomes, accounted for 43% of the total included patients in the meta-analysis. Therefore, if the study conducted by Lee et al. was excluded, it could severely weaken the validity of the meta-analysis results [18].

Two of the included studies reported only the survival or mortality of patients, and did not report the neurological outcome, which was defined as PNO [3,16]. Although a CPC score of 5 indicated patient mortality, patients with a CPC score of 3 or 4 in these studies were not included in the meta-analysis for PNO. These inaccurate criteria for PNO could be confounding factors affecting the results of the meta-analysis. In addition, the early neurological outcomes of PCAS at the time of discharge could be changeable based on the time of measurement until 6 months [30,31]. Therefore, a neurological outcome with a wide range of measurement time points, ranging from hospital discharge to six months, and failure to separate the outcome according to the measurement time points could have caused inaccurate results.

Hence, we performed a meta-analysis of the neurological outcomes and mortality separately according to the measurement time points [32,33], and we excluded any study that did not report the cut-off point, sensitivity, and specificity of ONSD. Additionally, one study was excluded because the location and timing of ONSD measurement were not recorded, and another study using MRI was also excluded for not being available routinely in emergency setting [2,4].

In this study, the measured ONSD was relatively higher in patients with PNO than in those with GNO. However, the high heterogeneity (I^2^ > 75%) and small number of included studies in the subgroups (at discharge, 1 month, 3 months, and 6 months) of this study made it difficult to determine whether ONSD is useful for predicting neurological outcomes. Although we performed subgroup, sensitivity, and meta-regression analyses to investigate the potential causes of heterogeneity, there was no substantial reduction in heterogeneity after these additional analyses.

This unsolved high heterogeneity can be explained by several factors. First, we analyzed the data of all modalities without considering the characteristics of the modality used for ONSD measurement, such as optic US and brain CT. An ONSD measurement using optic US is more reliable than using brain CT, because brain CT is not obtained parallel to the optic nerve sheath, thus leading to an oblique image rather than a horizontal image of the optic nerve sheath [8]. In contrast, in ocular US, the ONSD measurement value is obtained with the image perpendicular to the optic sheath, thus rendering a more accurate image of ONSD than that obtained using brain CT. As a result, adding some studies in the meta-analysis that employed brain CT could lead to more heterogeneity in the pooled outcomes than studies that exclusively used optic US. [3,16,19,21]. Second, we performed a meta-analysis of the results of studies that did not consider the effect of TTM on neurological outcomes. Among the seven included studies, three had a TTM rate of less than 50% (Table S2) [17,20,21]. This difference in the TTM rate may have affected the neurological outcomes of patients. Third, in healthy adults, there are differences in the baseline ONSD according to individual characteristics including sex, body mass index (BMI), race, or eyeball size [34,35,36]. Most studies conducted on healthy volunteers have demonstrated varying mean or median ONSD values across study cohorts, depending on race or measurement tools [37,38]. An US evaluation of healthy Asians revealed a higher ONSD value in men and individuals with a high BMI [34,36]. Therefore, these individual differences could also have confounded the interpretation of ONSD.

Finally, regarding ICP, there is an eye pressure test that clearly correlates eye pressure with ONSD in the study by Abegao Pinto et al. [39]; however, studies on PCAS have not presented the actual ICP of patients but only described the relationship between ICP and ONSD. Therefore, there is no way to identify if ONSD expansion is due to ICP elevation, if some patients have a naturally large diameter, or if there are other causes that led to ONSD expansion. We also cannot rule out the possibility that these limitations of the study may have caused heterogeneity, along with the type of measurement modality.

With respect to the prognostic accuracy of ONSD for the neurological outcomes, the study showed a significant pooled DOR with a lower heterogeneity (DOR = 23.97; 95% CI = 7.18–80.01; I^2^ = 0) than that in the previous meta-analysis conducted by Zhang et al. [8] (DOR = 15.62; 95% CI = 5.50–44.34; I^2^ = 58.4) (Table S7). The measurement time, which ranged from hospital discharge to 3 months, was also shorter than Zhang et al.’s, which ranged from hospital discharge to 6 months. This was due to the exclusion of two studies [3,16] for pooled DOR that were included in the meta-analysis by Zhang et al. but did not report ONSD as a neurological outcome.

### Limitations

We acknowledge several limitations in our large meta-analyses. First, all studies included in the present meta-analysis were observational studies with the potential for patient selection bias. This may have led to inappropriate eligibility criteria and no control over confounding factors. Second, most studies originated from Asia, potentially limiting the generalizability to other healthcare systems. The results of this study may have been different if cohorts from other countries or ethnicities were included. For more robust conclusions, further analyses should include populations from diverse countries. Third, there were heterogeneities between CA type (OHCA vs. IHCA) and cardiac origin (Table 1 and Table S2). Generally, patients with OHCA have worse neurological outcomes than those with IHCA. Furthermore, patients with CA of non-cardiac origin have worse neurological outcomes than patients with CA of cardiac origin [40]. This heterogeneity may have contributed to the neurological outcomes in patients with PCAS. To resolve this heterogeneity, data from future studies should be more detailed and categorized according to CA type and cause.

## 5. Conclusions

ONSD measurement might be an effective predictor for short-term PNO in PCAS. In the analysis by the measurement time of outcomes, there was no significant evidence to demonstrate that ONSD measurement was effective in predicting mortality and PNO.

## Figures and Tables

**Table 1:** Study characteristics.

Author	Year	Country	Inclusion Period	Study Type	Sample Size, CA Type	PNO (CPC 3–5), *n* (%)	Death, *n* (%)	ONSD Measurement			Outcome Measurement
								Modality	Location	Time after ROSC	Timepoint
Chae	2016	Korea	2009–2013	sROS	119	74 (62.2)	NR	CT	3 mm behind the globe	0–6 h	1 month
Chelly	2016	France	2011–2013	mPOS	36	22 (61.1) †	17 (47.2)	US	3 mm behind the globe	24 h	at hospital discharge
Ertl	2018	Germany	2015–2017	mPOS	49	NR	25 (51.0)	US	3 mm behind the globe	0–24 h	at hospital discharge
Kim	2014	Korea	2012–2013	sROS	91	68 (74.7)	NR	CT	3 mm behind the globe	0–24 h	at hospital discharge
Lee	2018	Korea	2015–2016	mPOS	329 *	230 (69.9)	167 (50.8)	CT	3 mm behind the globe	0–2 h	6 months
Park	2019	Korea	2018–2019	sPOS	36 *	18 (50.0)	NR	US	3 mm behind the globe	24 h	3 months
Rush	2017	Canada	2009–2013	sROS	72	54 (75.0)	53 (73.6) †	CT	3 mm behind the globe	0–48 h	at hospital discharge
Ryu	2017	Korea	2005–2015	sROS	42	23 (54.8)	19 (45.2) †	CT	3 mm behind the globe	0–48 h	at hospital discharge
Ueda	2015	Japan	2013–2014	sROS	17	11 (64.7) **	NR	US	3 mm behind the globe	12–72 h	1 month

Abbreviations: CA = cardiac arrest; PNO = poor neurologic outcome; ONSD = optic nerve sheath diameter; ROSC = return of spontaneous circulation; NR = not reported; CT = computed tomography; US = ultrasound; h = hour; CPC = cerebral performance category; sROS = single-center retrospective observational study; mPOS = multi-center prospective observational study; sPOS = single-center prospective observational study. * Out-of-hospital cardiac arrest patients were only included in the study. ** PNO was defined as Glasgow outcome scale (GOS) 1–3 in the study by Ueda 2015. † Not included in the meta-analysis because ONSD was not reported in the study.

## Data Availability

The datasets generated during the current study are available from the corresponding author on reasonable request.

## Supplementary Materials

The following supporting information can be downloaded at https://www.mdpi.com/article/10.3390/jpm12030500/s1. Table S1: Comprehensive list presenting the search strategy; Table S2: Patients’ characteristics; Table S3: Detailed analysis of prognostic accuracy of the optic nerve sheath diameter for poor neurologic outcomes in each study; Table S4: Subgroup analysis of included studies to identify the association of optic nerve sheath diameter with a poor neurologic outcome; Table S5: Sensitivity analysis of included studies to identify the association of optic nerve sheath diameter with the neurologic outcome; Table S6: Meta-regression analyses for potential causes of heterogeneity; Table S7: Analysis of prognostic accuracy for a poor neurologic outcome of this updated meta-analysis compared with previous meta-analyses; Table S8: GRADE profile for assessing quality of evidence for the included studies for outcomes; Figure S1: Assessment of study quality; Figure S2: Pooled prognostic accuracy of the optic nerve sheath diameter for poor neurological outcome.
