# Supplementary material for: Optic Nerve Sheath Diameter for Predicting Outcomes in Post-Cardiac Arrest Syndrome: An Updated Systematic Review and Meta-Analysis
Source: J Pers Med. 2022 Mar 20;12(3):500. doi: 10.3390/jpm12030500 (PMC8953152; doi:10.3390/jpm12030500)
Supplement: Supplementary file 1 [file jpm-12-00500-s001.zip › jpm-1558986-supplementary.pdf]

Supplementary Material

Table S1. Comprehensive list presenting the search strategy.

Embase.

No.	Searches	Results
1	(return of spontaneous circulation or ROSC).ti,ab. or exp heart arrest/ or cardiac arrest*.ti,ab. or cardiovascular arrest*.ti,ab. or heart arrest*.ti,ab. or cardiopulmonary arrest*.ti,ab. or asystol*.ti,ab. or pulseless electrical activity.ti,ab. or exp ventricular fibrillation/ or exp advanced cardiac life support/ or (advanced cardiac life support or ACLS).ti,ab. or exp cardiopulmonary resuscitation/ or CPR.mp. or cardiopulmonary resuscitation.ti,ab.	224,915
2	optic nerve sheath diameter.mp. or exp optic nerve/ or exp optic nerve sheath diameter/ or nerve sheath/	27,533
3	ONSD.ti,ab.	37
4	2 or 3	27,533
5	1 and 4	87

Medline.

No.	Searches	Results
1	(return of spontaneous circulation or ROSC).ti,ab. or exp heart arrest/ or cardiac arrest*.ti,ab. or cardiovascular arrest*.ti,ab. or heart arrest*.ti,ab. or cardiopulmonary arrest*.ti,ab. or asystol*.ti,ab. or pulseless electrical activity.ti,ab. or exp ventricular fibrillation/ or exp advanced cardiac life support/ or (advanced cardiac life support or ACLS).ti,ab. or exp cardiopulmonary resuscitation/ or CPR.mp. or cardiopulmonary resuscitation.ti,ab.	98,122
2	exp Optic Nerve/ or optic nerve sheath diameter.mp.	31,556
3	ONSD.ti,ab.	11
4	2 or 3	31,556
5	1 and 4	41

Cochrane library.

No.	Searches	Results
1	MeSH descriptor: (Return of Spontaneous Circulation) explode all trees	1
2	MeSH descriptor: (Heart Arrest) explode all trees	1998
3	MeSH descriptor: (Resuscitation) explode all trees	5177
4	MeSH descriptor: (Advanced Cardiac Life Support) explode all trees	60
5	1 or 2 or 3 or 4	6463
6	MeSH descriptor: (Optic Nerve) explode all trees	332
7	(ONSD):ti,ab,kw (Word variations have been searched)	83
8	6 or 7	399
9	5 and 8	0

Table S2. Patients' characteristics.

	Outcome	N	Age	Male, %	Cardiac origin, %	Witness, %	Bystander CPR, %	Shockable, %	CPR			After ROSC	
									No flow time, min	Low flow time, min	ROSC, min	Time of image modality, min	TTM %
Chae 2016	GNO	45	48.8 (16.3)	71.1	88.9	-	53.3	55.6	4 (5.8)	20.6 (17.9)	24.6 (18.6)	55.5 (35.5–121.5)	100
	PNO	74	56.4 (17.4)	55.4	36.5	-	35.1	20.3	12.7 (13.2)	26.4 (15.0)	39.0 (20.7)	63 (39–125)	100
	Total	119	53.53 (17.32)	61.3	-	-	-	33.6	-	-	-	-	100
Chelly 2016	Survival	19	53 (40–65)	68	53	90	79	63	1 (1–2)	10 (3-16)	-	-	-
	Death	17	58 (55–73)	71	18	65	41	18	9 (2–10)	20 (10-32)	-	-	-
	Total	36	58 (45–69)	69	36	78	64	42	2 (1–9)	15 (7-30)	-	-	-
Ertl 2018	Survival	23	59 (20–90)	74	-	-	-	74	-	-	-	-	77
	Death	26	69 (35–96)	69	-	-	-	46	-	-	-	-	46
	Total	49	65 (20–96)	71	-	-	-	59	-	-	-	-	60
Kim 2014	GNO	23	50 (44–59)	74	61	91	61	52	-	-	11 (5–25)	60 (21–149)	56
	PNO	68	61 (48–71)	59	31	34	29	15	-	-	31.5 (20.5–48)	55 (30–120)	40
	Total	91	57.73 (16.60)	62.6	-	-	-	24.2	-	-	-	-	-
Lee 2018	GNO	99	52.03 (14.41)	71.72	84.85	79.80	72.73	60.61	2.42 (4.35)	21.28 (13.68)	-	-	100
	PNO	230	61.47 (15.92)	69.13	51.74	62.61	59.13	14.35	4.58 (7.56)	29.07 (14.93)	-	-	100
	Survival	162	54.61 (15.69)	65.42	71.60	73.46	66.67	43.83	2.79 (4.43)	22.14 (13.07)	-	-	100
	Death	167	62.64 (15.44)	71.86	52.10	62.28	59.88	13.17	5.04 (8.39)	31.19 (15.40)	-	-	100
	Total	329	58.63 (16.06)	69.9	-	-	-	28.3	-	-	-	-	100
Park 2019	GNO	18	50.67 (15.49)	83.33	50.00	72.22	88.89	61.11	0.5 (0–5.0)	12.67 (9.05)	-	-	100
	PNO	18	53.00 (15.93)	61.11	11.12	50.00	61.11	11.12	3.5 (0.75–16.25)	27.50 (13.68)	-	-	100
	Total	36	51.83 (15.53)	72.22	30.56	61.11	75.00	14.11	2.0 (0-12.75)	20.08 (13.68)	-	-	100
Rush 2017	GNO	18	57.7 (15.3)	83.3	-	94.4	88.9	38.9	-	-	9.3 (7.8)	9.3 (10.0)	-
	PNO	54	64.1 (15.1)	83.3	-	76.0	79.6	27.8	-	-	27.7 915.0)	10.2 (11.2)	-
	Total	72	62.5 (15.3)	83.3	-	-	-	30.6	-	-	-	-	-
Ryu 2017	GNO	19	41.0 (32.0–70.5)	73.7	-	100	100	42.1	-	-	-	-	36.8
	PNO	23	52.0 (43.0–60.5)	65.2	-	100	100	39.1	-	-	-	-	39.1
	Total	42	51.0 (34.0–65.0)	69.0	-	100	100	40.5	-	-	-	-	38.1
Ueda 2015	Total	17	74.8 (55–92)	53	-	-	71	29	-	-	-	-	24

Data are presented as mean (SD) or median (interquartile range).

Table S3. Detailed analysis of prognostic accuracy of the optic nerve sheath diameter for poor neurologic outcomes in each study.

Study	ONSD cut-off point (mm)	N	TP (n)	FP (n)	FN (n)	TN (n)	Sensitivity (%)	Specificity (%)	PPV (%)	NPV (%)
Chae	7.0	119	4	0	70	45	5.5	100	100	38.4
Kim	6.21	91	38	0	30	23	55.9	100	100	43.4
Park	4.9	36	15	1	3	17	83.3	94.4	93.8	85.0
Ryu	6.69	42	5	0	18	19	21.7	100	100	51.4
Ueda	5.4	17	8	1	3	5	72.7	83.3	88.9	62.5

ONSD = optic nerve sheath diameter; TP = true positive; FP = false positive; FN = false negative; TN = true negative; PPV = positive predictive value; NPV = negative predictive value.

Supplementary Table S4. Subgroup analysis of included studies to identify the association of optic nerve sheath diameter with poor neurologic outcome.

Covariate	*N*	ONSD, SMD (95% CI)	*p*-value	I^2, %	*p*-value for heterogeneity
All	7	0.74 (0.22, 1.27)	0.006	87	<0.001
Country					
South Korea	5	0.93 (0.27, 1.60)	0.006	91	<0.001
Other countries	2	0.15 (–0.5, 0.80)	0.65	33	0.22
PNO, %					
> 65	3	0.56 (–0.4, 1.51)	0.25	93	<0.001
< 65	4	0.91 (0.21, 1.62)	0.01	78	0.004
Modality					
CT	5	0.55 (0.01, 1.09)	0.05	88	<0.001
US	2	1.37 (0.03, 2.71)	0.04	76	0.04
TTM					
100%	3	0.7 (–0.04, 1.44)	0.06	90	<0.001
<100%	3	1.11 (0.43, 1.79)	0.001	65	0.06

N = the number of studies; ONSD = optic nerve sheath diameter; SMD = standardized mean differences; CI = confidence interval; PNO = poor neurologic outcome; CT = computed tomography; MRI = magnetic resonance imaging; US = ultrasound; TTM = targeted temperature management.

Table S5. Sensitivity analysis of included studies to identify the association of optic nerve sheath diameter with neurologic outcome.

Study	ONSD, SMD (95% CI)	*p*-value	I^2, %	*p*-value for heterogeneity
All	0.74 (0.22, 1.27)	0.006	87	< 0.001
Omitting Chae 2016	0.83 (0.15, 1.51)	0.02	89	<0.001
Omitting Kim 2014	0.55 (0.09, 1.00)	0.02	79	0.0002
Omitting Lee 2018	0.88 (0.24, 1.52)	0.007	85	< 0.001
Omitting Park 2019	0.56 (0.07, 1.05)	0.03	85	< 0.001
Omitting Rush 2017	0.89 (0.29, 1.49)	0.004	89	< 0.001
Omitting Ryu 2017	0.74 (0.14, 1.34)	0.02	89	< 0.001
Omitting Ueda 2015	0.76 (0.18, 1.33)	0.01	89	< 0.001

ONSD = optic nerve sheath diameter; SMD = standardized mean differences; CI = confidence interval.

Table S6. Meta-regression analyses for potential causes of heterogeneity.

Covariates	*N*	*p*-value	Regression coefficient (ß)
Sample size	7	0.2510	–0.0031
Time from ROSC to ONSD measurement	7	0.9824	0.0003
Age	7	0.4006	–0.0336
Male, %	7	0.5100	–0.0212
Shockable rhythm, %	7	0.0805	–0.0624

N = the number of studies; ROSC = return of spontaneous circulation; ONSD = optic nerve sheath diameter.

Table S7. Analysis of prognostic accuracy for poor neurologic outcome of this updated meta-analysis comparing with previous meta-analyses.

	This updated meta-analysis (total *N*)						Previous meta-analyses (total *N*)			
	**3 CT + 2 US (5)**	**I^2 (%)**	**3 CT (4)**	**I^2 (%)**	**2 US (2)**	**I^2 (%)**	**5 CT + 3 US (8) (Lee 2019)**	**I^2 (%)**	**3 CT + 5 US (8) (Zhang 2020)**	**I^2 (%)**
**Pooled SEN**	0.361 (0.293–0.433)	94.7	0.285 (0.217–0.360)	95.9	0.793 (0.603–0.920)	0	0.41 (0.20–0.67),	95.7	0.60 (0.45–0.73)	-
**Pooled SPE**	0.982 (0.936–0.998)	42.0	1.000 (0.958–1.000)	0	0.917 (0.730–0.990)	0	0.99 (0.82–1.0),)	94.6	0.94 (0.83–0.98)	-
**Pooled PLR**	9.097 (3.258–25.402)	0	11.382 (2.227–58.175)	0	7.852 (2.094–29.442)	0	49.0 (2.4–958.9)	-	-	-
**Pooled NLR**	0.504 (0.254–1.002)	96.9	0.703 (0.397–1.245)	96.5	0.241 (0.116–0.501)	0	0.59 (0.39–0.90)	-	-	-
**DOR**	23.971 (7.182–80.008)	0	16.408 (3.048–88.322)	0	35.527 (5.799–217.64)	9.2	83 (4–1525)	-	15.62 (5.50–44.34)	-
**SROC (AUC)**	0.8668	-	0.7395	-	0.5000	-	0.86 (0.83–0.89)*	-	0.87 (0.84–0.90)	-
**SE (AUC)**	0.0776	-	0.0882	-	0	-	-	-	-	-
**Q**	0.7973	-	0.6848	-	0.5	-	-	-	-	-
**SE (Q)**	0.0760	-	0.0723	-	0	-	-	-	-	-

N = the number of studies; ROSC = return of spontaneous circulation; ONSD = optic nerve sheath diameter; CT = computed tomography; US = ultrasonography; SEN = sensitivity; SPE = specificity; PLR = positive likelihood ratio; NLR = negative likelihood ratio; DOR = diagnostic odds ratio; SROC = summary receiver operating characteristic; SE = standard error; AUC = area under the curve; CI = confidence interval; ONSD = optic nerve sheath diameter; LR = likelihood. * hierarchical summary receiver operating characteristic (HSROC).

Table S8. GRADE profile for assessing quality of evidence for the included studies for outcomes

Author(s):
Question: Optic nerve sheath diameter for predicting outcomes in post-cardiac arrest syndrome
Setting:
Bibliography:

Certainty assessment							№ of patients		Effect		Certainty	Importance
№ of studies	Study design	Risk of bias	Inconsistency	Indirectness	Imprecision	Other considerations	ONSD of PNO or death	ONSD of GNO or survival	Relative (95% CI)	Absolute (95% CI)		
**Poor neurological outcome at hospital discharge**												
3	observational studies	not serious	serious[a]	not serious	serious[b]	none	145	60	-	SMD **0.8 SD higher** (0.25 lower to 1.85 higher)	⊕⊕◯◯ Low	CRITICAL
**Poor neurological outcome at 1 month**												
2	observational studies	not serious	serious[c]	not serious	serious[b]	none	85	51	-	SMD **0.39 SD higher** (0.04 higher to 0.74 higher)	⊕⊕◯◯ Low	CRITICAL
**Poor neurological outcome at 3 months**												
1	observational studies	serious[d]	serious	not serious	serious[b]	none	18	18	-	SMD **2.02 SD higher** (1.2 higher to 2.84 higher)	⊕◯◯◯ Very low	CRITICAL
**Poor neurological outcome at 6 months**												
1	observational studies	serious[e]	not serious	not serious	serious[f]	none	230	99	-	SMD **0.11 SD higher** (0.13 lower to 0.34 higher)	⊕⊕◯◯ Low	CRITICAL
**Death at hospital discharge**												
2	observational studies	serious[e]	not serious	serious[g]	serious[b]	none	42	43	-	SMD **1.28 SD higher** (0.81 higher to 1.75 higher)	⊕◯◯◯ Very low	CRITICAL
**Death at 6 months**												
1	observational studies	not serious	not serious	not serious	serious[f]	none	167	162	-	SMD **0.12 SD higher** (0.09 lower to 0.34 higher)	⊕◯◯◯ Very low	CRITICAL

CI: confidence interval; **SMD:** standardised mean difference

Explanations

a. Neither the same direction nor similar magnitude of the effect
b. Total sample size limited
c. The studies had a wide confidence interval spanning.
d. High flow and timing
e. High index test
f. The total included studies are too small
g. the only short-term outcome was measured.

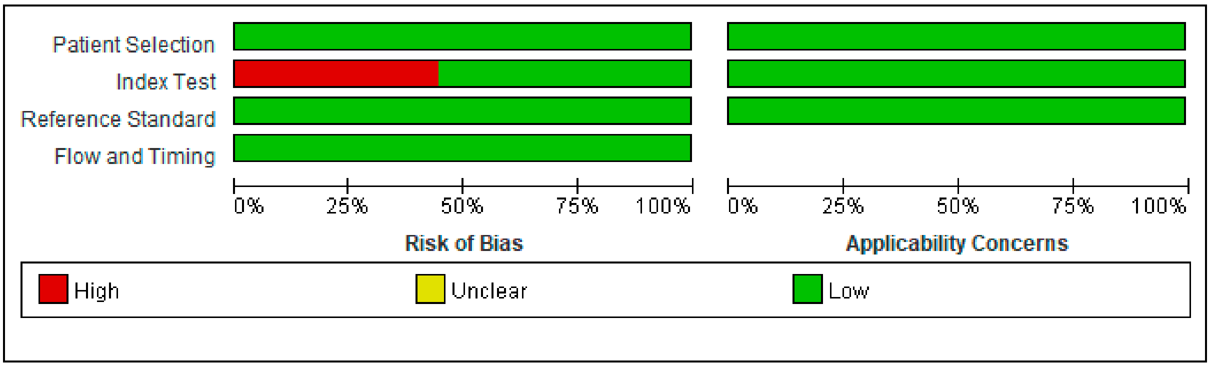

(a)

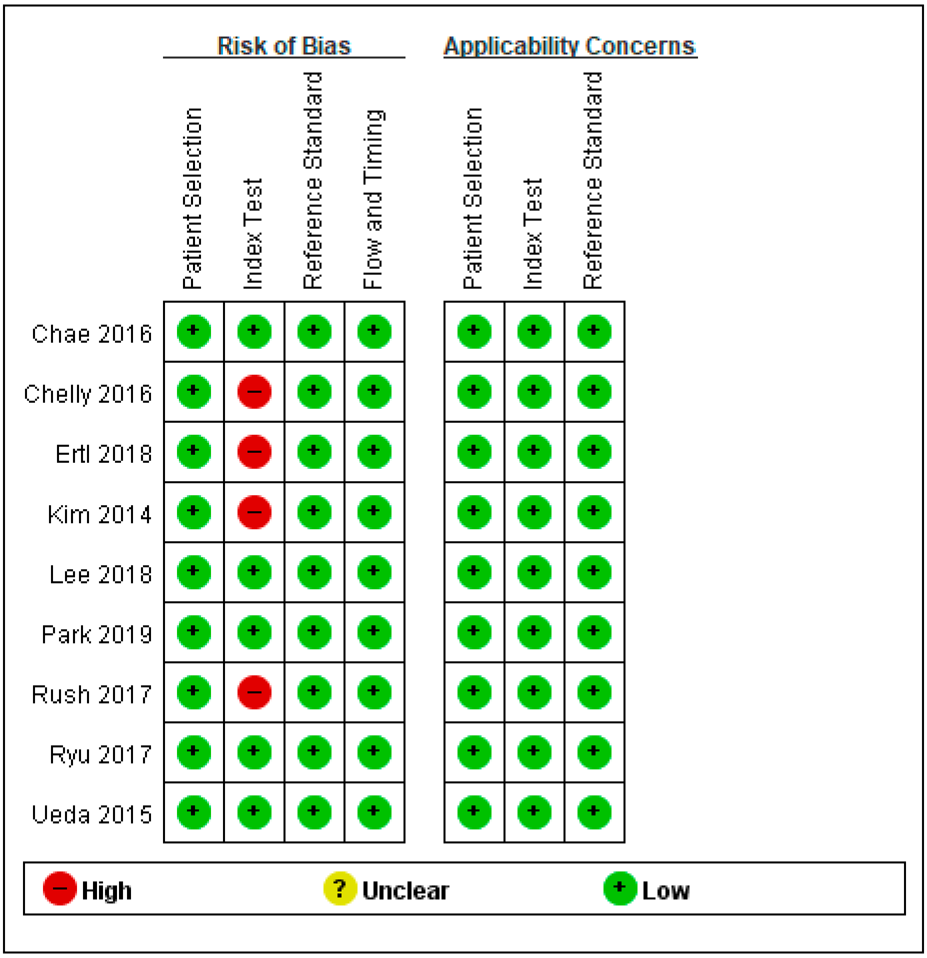

(b)

Figure S1. Assessment of study quality.

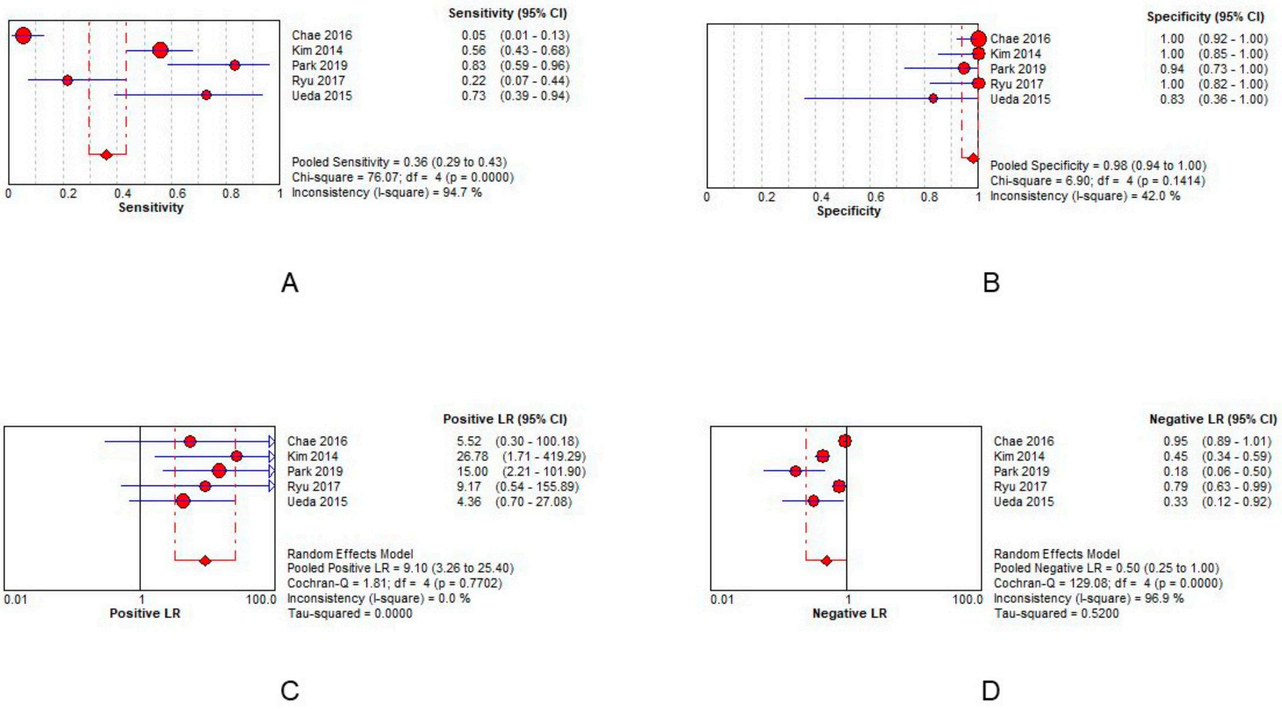

Figure S2. Pooled prognostic accuracy of the optic nerve sheath diameter for poor neurological outcome. **A**: Pooled sensitivity, **B**: pooled specificity, **C**: pooled positive likelihood ratio, **D**: pooled negative likelihood ratio. Abbreviations: CI = confidence interval; LR = likelihood.
